# Effect of empagliflozin on ketone bodies in patients with stable chronic heart failure

**DOI:** 10.1186/s12933-021-01410-7

**Published:** 2021-11-09

**Authors:** R. Pietschner, J. Kolwelter, A. Bosch, K. Striepe, S. Jung, D. Kannenkeril, C. Ott, M. Schiffer, S. Achenbach, R. E. Schmieder

**Affiliations:** 1 Department of Nephrology and Hypertension, University Hospital Erlangen, Friedrich-Alexander-Universität Erlangen-Nürnberg (FAU), Ulmenweg 18, 91054 Erlangen, Germany; 2 Department of Cardiology, University Hospital Erlangen, Friedrich-Alexander-Universität Erlangen-Nürnberg (FAU), Erlangen, Germany; 3 Department of Nephrology and Hypertension, Paracelsus Medical University, Nuremberg, Germany

## Abstract

**Background:**

Recent studies indicated that sodium glucose cotransporter (SGLT)2 inhibition increases levels of ketone bodies in the blood in patients with type 1 and 2 diabetes. Other studies suggested that in patients with chronic heart failure (CHF), increased myocardial oxygen demand can be provided by ketone bodies as a fuel substrate. Experimental studies reported that ketone bodies, specifically beta-hydroxybutyrate (β-OHB) may increase blood pressure (BP) by impairing endothelium-dependant relaxation, thereby leading to increased vascular stiffness. In our study we assessed whether the SGLT 2 inhibition with empagliflozin increases ketone bodies in patients with stable CHF and whether such an increase impairs BP and vascular function.

**Methods:**

In a prospective, double blind, placebo controlled, parallel-group single centre study 75 patients with CHF (left ventricular ejection fraction 39.0 ± 8.2%) were randomised (2:1) to the SGLT-2 inhibitor empagliflozin 10 mg orally once daily or to placebo, 72 patients completed the study. After a run-in phase we evaluated at baseline BP by 24 h ambulatory blood pressure (ABP) monitoring, vascular stiffness parameters by the SphygmoCor system (AtCor Medical, Sydney, NSW, Australia) and fasting metabolic parameters, including β-OHB by an enzymatic assay (Beckman Coulter DxC 700 AU). The same measurements were repeated 12 weeks after treatment. In 19 of the 72 patients serum levels of β-OHB were beneath the lower border of our assay (< 0.05 mmol/l) therefore being excluded from the subsequent analysis.

**Results:**

In patients with stable CHF, treatment with empagliflozin (n = 36) was followed by an increase of β-OHB by 33.39% (p = 0.017), reduction in 24 h systolic (p = 0.038) and diastolic (p = 0.085) ABP, weight loss (p = 0.003) and decrease of central systolic BP (p = 0.008) and central pulse pressure (p = 0.008). The increase in β-OHB was related to an attenuated decrease of empagliflozin-induced 24 h systolic (r = 0.321, p = 0.069) and diastolic (r = 0.516, p = 0.002) ABP and less reduction of central systolic BP (r = 0.470, p = 0.009) and central pulse pressure (r = 0.391, p = 0.033). No significant changes were seen in any of these parameters after 12 weeks of treatment in the placebo group (n = 17).

**Conclusion:**

In patients with stable CHF ketone bodies as assessed by β-OHB increased after treatment with empagliflozin. This increase led to an attenuation of the beneficial effects of empagliflozin on BP and vascular parameters.

*Trial registration* The study was registered at http://www.clinicaltrials.gov (NCT03128528).

## Introduction

Sodium glucose cotransporter (SGLT)2 inhibitors introduced as antidiabetic agents have been found to decrease incidence of cardiovascular mortality, heart failure hospitalisations and renal events of progressive renal disease [1]. Their organo-protective effects have been found in patients with as well as without type 2 diabetes (T2D) [2, 3]. SGLT2 inhibition represents nowadays a novel treatment of chronic heart failure (CHF) with outlooks on even more possible treatment indications [4]. Yet, the mediators of these improved outcomes remain to be determined.

Blood levels of ketones have been described to be increased in patients with type 1 and 2 diabetes after treatment with SGLT2 inhibitors [5, 6]. A recent study, called “the fuel hypothesis”, attributed the cardiovascular benefits of SGLT2 inhibition to improved energetics by a metabolism shift towards increased ketosis. According to this concept, ketone body fuel utilization would improve transduction of oxygen into increased work efficiency [7]. In patients with CHF, it is believed that the capacity of the myocardium to use free fatty acids is diminished and a shift to ketone body utilization as fuel for oxidative ATP production takes place [8]. In a recent interventional study, patients with CHF were treated with β-hydroxybutyrate (β-OHB) infusions. β-OHB showed beneficial hemodynamic effects by increasing cardiac output and left ventricular ejection fraction, without impairing myocardial efficiency [9]. Similar observations have been made in patients with T2D indicating that the myocardium of patients with T2D prefers ketones as an energy fuel source [10]. Furthermore, antiinflammatory and antioxidative properties of SGLT2 inhibition in patients with T2D have been shown to be related to β-OHB increases [11]. These findings support a potential predominant role of β-OHB for the mediation of SGLT2 inhibitors-related organoprotective effects. Whereas ketone bodies have been already analysed in several animal models and humans after acute myocardial infarction under therapy with SGLT2 inhibition, there are no data on ketone bodies in a stable situation of patients with CHF after SGLT2 inhibitor therapy.

The aim of the current analysis is to assess the influence of the SGLT2 inhibitor empagliflozin on blood levels of ketone bodies in patients with CHF and to analyse their relation to blood pressure (BP) and to vascular function.

## Methods

### Study design

This was a secondary analysis of an investigator initiated, prospective, double blind, placebo controlled, parallel-group phase II single centre study in order to analyse changes in blood β-OHB levels in patients with CHF following SGLT2 inhibition with empagliflozin. Furthermore, the relationship between β-OHB and parameters of vascular stiffness were analysed. All participants were treated in our Clinical Research Unit of the Department of Nephrology and Hypertension, University of Erlangen-Nuremberg, Germany (http://www.clinicaltrials.gov: NCT03128528). Between July 2017 and March 2020, subjects with stable CHF were recruited from the investigator’s outpatient clinics, referring physicians, and advertisement in local newspapers, and social media. After being off any SGLT-2 inhibitors for at least 10 weeks, subjects entered a run-in phase of 2 weeks and primary and secondary evaluation criteria were assessed. Patients were then consecutively randomised to either empagliflozin 10 mg orally once daily or placebo (2:1) for 12 weeks. The study was approved by the local Ethics Committee (University of Erlangen-Nuremberg) and performed in accordance with the Declaration of Helsinki and the principles of good clinical practice guidelines.

### Study population

Subjects between 18 and 85 who had heart failure with mid-range ejection fraction (HFmEF) in stable conditions or heart failure with reduced ejection fraction (HFrEF) in stable conditions, according to the ESC guidelines for the diagnosis and treatment of acute and chronic heart failure were included in the study [12, 13]. Patients who had heart failure with preserved ejection fraction (LVEF ≥ 50%) were not included in the study. Other key exclusion criteria were any other form of diabetes than T2D, treatment with insulin or any SGLT-2 inhibitors within the past 10 weeks prior to screening, a HbA1c ≥ 10% or fasting plasma glucose ≥ 240 mg/dl, an estimated glomerular filtration rate < 30 ml/min/1.73 m^2^, uncontrolled hypertension, congestive heart failure New York Heart Association IV or the use furosemide > 80 mg/day, or torasemide > 40 mg/day, or piretanide > 6 mg/day. Any history of stroke, transient ischemic attack, instable angina pectoris or acute myocardial infarction within the last 6 months prior to study inclusion led to an exclusion of the study.

### Clinical parameters

Demographic data were recorded at the first visit (screening). At randomisation (baseline), fasting blood samples were withdrawn in order to evaluate HbA1c, fasting plasma glucose, lipid levels, β-OHB levels (our primary endpoint of this secondary analysis) and other bio-chemical safety parameters (e.g. creatinine, liver enzymes). Blood levels of β-OHB, were measured by an automated clinical chemistry analyser (Beckman Coulter DxC 700 AU), with a lower detection border for β-OHB of 0.05 mmol/l and a variation coefficient 1.68% for normal controls and—2.29% for pathological controls. Squared average of the errors of measurement was 0.00802 with an error margin of 0.02271.

Office brachial BP was assessed according to the European Society of Hypertension guideline recommendations by an oscillometric device (Dinamap Pro 100V2; Criticon, Norderstedt, Germany) and averages of the last three measurements were taken [14].

### Vascular function

#### Central blood pressure

Central (aortic) BP represents the BP acting directly on internal organs like heart and kidney and have been found to independently predict cardiovascular outcome [15]. A validated system (SphygmoCor XCEL System; AtCor Medical, Sydney, Australia) was used to derive the central systolic BP (cSBP) and central pulse pressure (cPP) [16]. Brachial artery pressure curves were recorded from the brachial artery, with the patient being in a supine position, using a high-fidelity applanation tonometer (Millar Instruments, Houston, Tex.), directly into the SphygmoCorTM System which then calculated the corresponding central aortic waveform. After previous calibration, corresponding central (aortic) waveforms were automatically calculated from the brachial artery waveform by a validated transfer function. From the derived central waveforms, data for cSBP, cPP, forward and backward wave amplitude were provided.

#### Ambulatory blood pressure monitoring

Twenty four-hour ambulatory BP (ABP) monitoring was assessed by a repeatedly validated device (Mobil-O-Graph, I.E.M., Aachen, Germany) [17]. BP measurements were taken at an interval of 15 min during the day and every half an hour during the night.

### Statistical analysis

Before further analysis, abnormal or normal distribution of variables was evaluated using Kolmogorov–Smirnov test. Normally distributed data are expressed as mean ± standard deviation and not normally distributed data presented as median and interquartile range (IQR). A two-sided P-value < 0.05 was considered statistically significant. The comparison between baseline and the end of the 12 week treatment phase within each treatment group was performed using the non-parametric Wilcoxon test (paired analysis). Statistical significance of differences between the empagliflozin and the placebo treatment arm was determined applying the non-parametric Mann–Whitney-test (unpaired analysis), accordingly. Correlations were calculated by using the Spearman analysis. All analyses were performed using SPSS software, version 24.0.0.2 (IBM Corporation, Chicago, IL, USA).

## Results

### Clinical characteristics of the study population

**Fig. 1:**
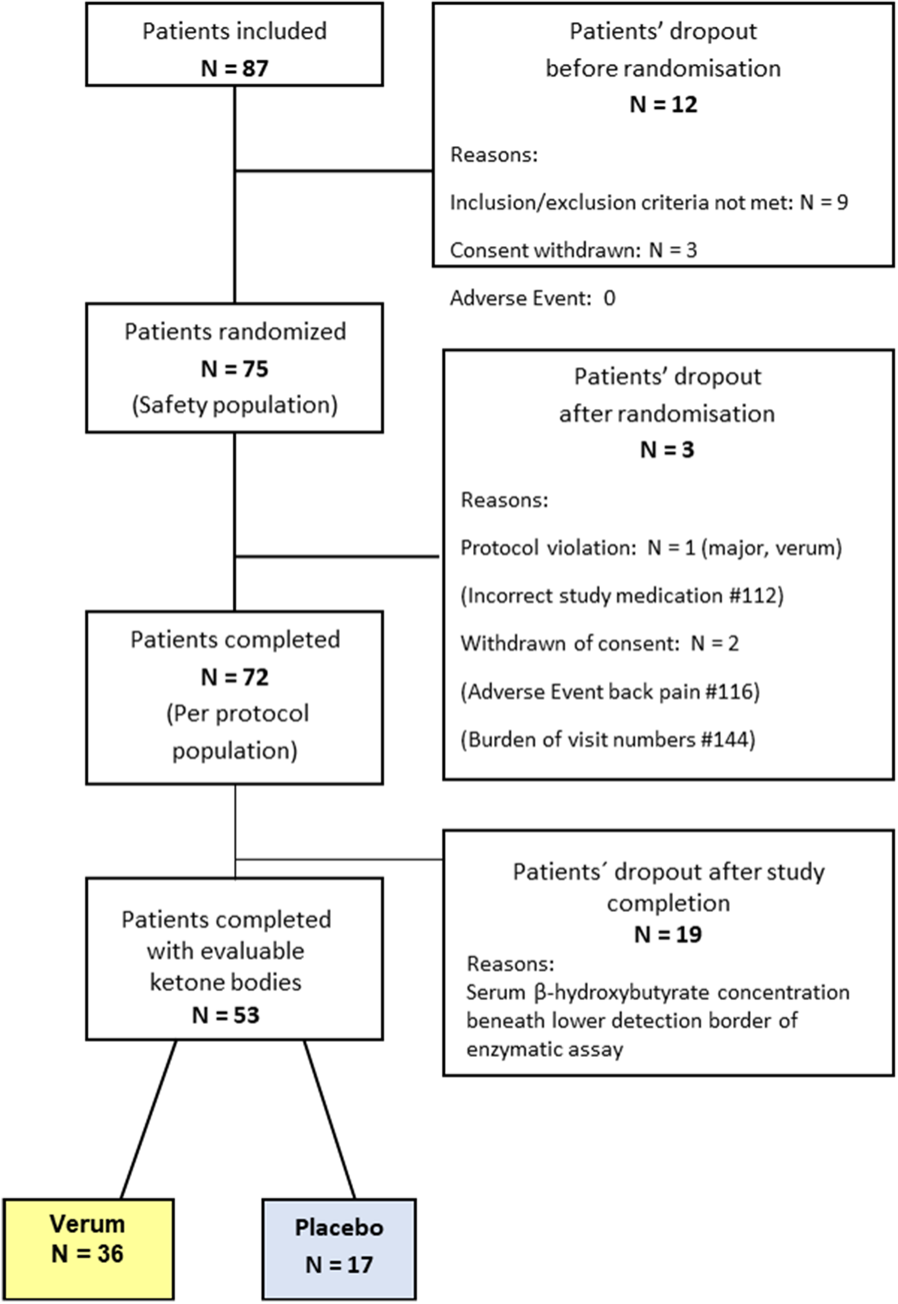
Patient disposition

A total of 87 patients with CHF were screened and 72 of them completed the study. In 19 patients at the time of both measurements (at baseline and 12 weeks after randomisation) levels of β-OHB were beneath the lower detection border of our assay (< 0.05 mmol/l) at baseline and 12 weeks after randomisation, therefore being excluded from the subsequent analysis (Empa: N = 12, Placebo: N = 6) (Fig. 1). The mean age of our study population N = 53 (Table 1) was 68.5 ± 8.2 years, 84.9% of them were male. Thirty-two patients had HFmEF, representing 61.5% percent of our study population with myocardial ischemia being the main cause of the CHF. Median NT-pro-BNP levels were 506.0 (IQR: 268.5–1311.0) pg/ml. Ejection fraction at baseline was 38.8 ± 8.6%, 79.2% had arterial hypertension and 24.5% T2D.

**Table 1:** Clinical characteristics of the study population

Parameter	All (N = 53)	Empa (N = 36)	Placebo (N = 17)
Age (years)	68.5 ± 8.2	69.0 ± 8.1	67.4 ± 8.7
Male sex [no, (%)]	45 (84.9)	29 (80.6)	16 (94.1)
Weight (kg)	88.7 ± 13.1	87.6 ± 13.8	90.8 ± 11.3
BMI (kg/m^2^)	28.9 ± 3.8	28.7 ± 4.0	29.2 ± 3.3
Office heart rate (bpm)	66.2 ± 12.2	65.8 ± 12.8	67.0 ± 11.3
Office systolic BP (mmHg)	124.5 ± 19.7	126.3 ± 20.0	120.5 ± 18.9
Office diastolic BP (mmHg)	72.4 ± 9.3	72.1 ± 9.6	72.9 ± 9.0
Left ventricular ejection fraction (%)	38.8 ± 8.6	39.8 ± 8.3	36.8 ± 9.1
NT-pro-BNP (pg/ml)	506.0 (268.5–1311.0)	499.0 (293.3–1401.8)	511.0 (189.0–1194.5)
Hypertension [no, (%)]	42 (79.2)	27 (75)	15 (88.2)
Type 2 diabetes [no, (%)]	13 (24.5)	10 (27.8)	3 (17.6)

Data are given as mean ± SD, NT-pro-BNP is given as median and interquartile range

*BMI* body mass index, *bpm* beats per minute, *BP* blood pressure, *NT-pro-BNP* N-terminal prohormone of brain natriuretic peptide

### Effect of empagliflozin treatment

**Fig. 2:**
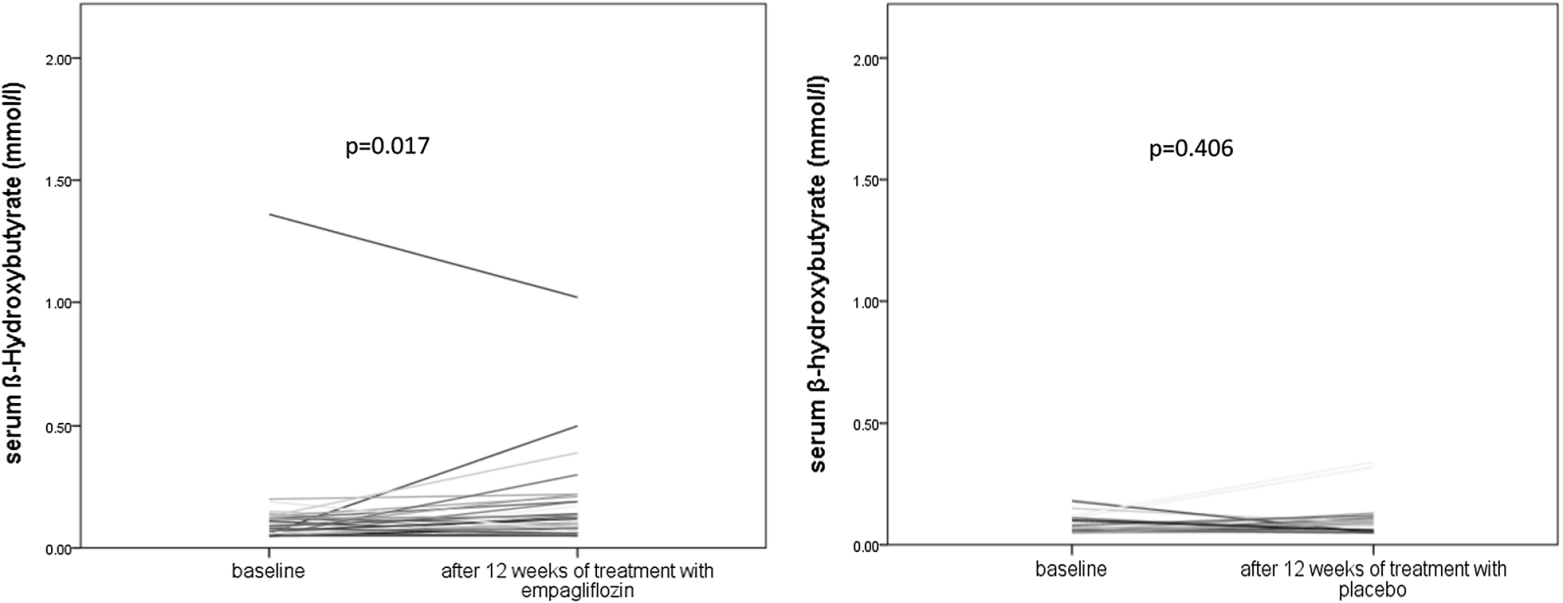
Change in β-hydroxybutyrate (β-OHB) between baseline and after 12 weeks of treatment with empagliflozin (left side) and between baseline and after 12 weeks of treatment with placebo (right side)

**Fig. 3:**
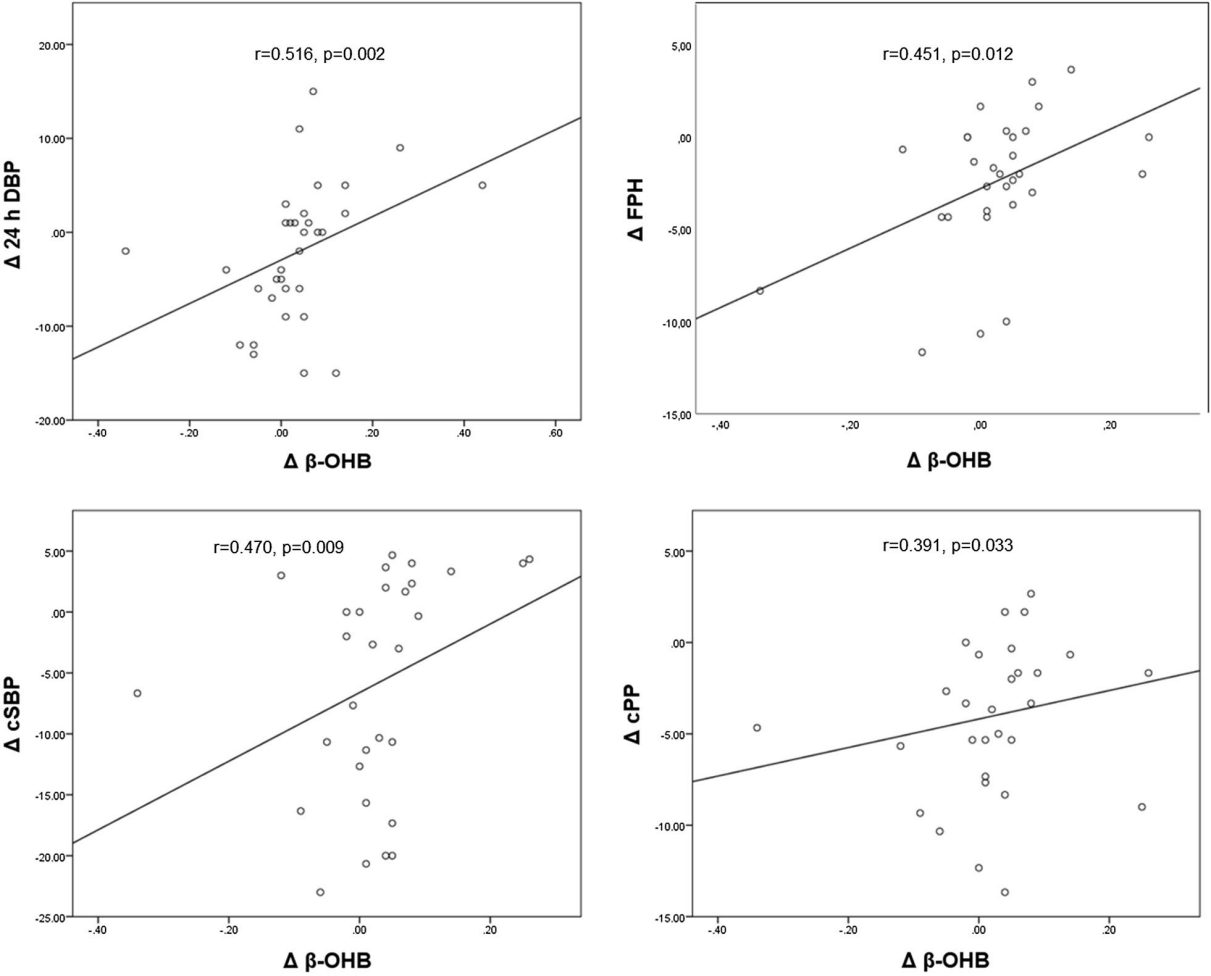
Relationship between changes in β-hydroxybutyrate, blood pressure and vascular parameters. Correlation between changes (Δ) in β-hydroxybutyrate (β-OHB) between baseline and after 12 weeks of treatment with empagliflozin and changes in 24 h diastolic blood pressure (24 h DBP) (top left side), in forward pulse pressure height (FPH) (top right side) in central systolic blood pressure (cSBP) (bottom left side) and in central pulse pressure (cPP) (bottom right side) between baseline and after 12 weeks of treatment with empagliflozin

Twelve weeks of treatment with empagliflozin was associated with an increase in β-OHB from 0.12 ± 0.22 mmol/l to 0.16 ± 0.18 mmol/l (p = 0.017) compared to baseline. There was no significant change in β-OHB after 12 weeks of treatment with placebo compared to baseline (from 0.08 ± 0.04 mmol/l to 0.11 ± 0.09 mmol/l, p = 0.406). Change of β-OHB between baseline and 12 weeks of treatment with empagliflozin was not significantly different compared to change between baseline and 12 weeks of treatment with placebo (p = 0.321) (Figs. 2 and 3). Change of β-OHB between baseline and 12 weeks of treatment with empagliflozin in patients with T2D was not significantly different compared to change of β-OHB between baseline and 12 weeks of treatment with empagliflozin in patients without T2D (data not shown).

Twelve weeks of treatment with empagliflozin was associated with a decrease in 24 h systolic ABP (p = 0.038), as well as in systolic (p = 0.005) and diastolic brachial (p = 0.269) BP compared to baseline (Table 2). Analysing vascular function, 12 weeks treatment with empagliflozin was associated with a significant decrease in cSBP (p = 0.008), cPP (p < 0.001) and forward pulse pressure height (p = 0.001) compared to baseline. There was no significant change in BP and vascular parameters after 12 weeks placebo compared to baseline (Table 2).

**Table 2:** Changes in blood pressure and vascular parameter

Parameter	n	Empa baseline	Empa 12 weeks	Change	p-value	n	Placebo baseline	Placebo 12 weeks	Change	p-value
Brachial systolic BP (mmHg)	30	131.4 ± 18.2	124.1 ± 12.9	− 6.8 ± 10.9	0.005	16	130.3 ± 22.0	129.2 ± 16.6	− 1.1 ± 16.5	0.796
Brachial diastolic BP (mmHg)	30	75.4 ± 10.1	73.3 ± 9.0	− 1.9 ± 7.9	0.269	16	76.0 ± 12.6	75.3 ± 8.7	− 0.7 ± 9.5	0.877
Brachial heart rate (bpm)	30	63.1 ± 10.2	63.3 ± 13.7	− 0.5 ± 8.3	0.650	16	60.0 ± 9.3	58.1 ± 12.6	− 1.9 ± 8.0	0.201
Systolic 24 h ABP (mmHg)	33	120.8 ± 16.2	117.5 ± 12.0	− 4.3 ± 11.6	0.038	14	122.1 ± 15.1	128.3 ± 18.1	+ 4.7 ± 8.6	0.084
Diastolic 24 h ABP (mmHg)	33	72.2 ± 9.4	70.9 ± 7.6	− 2.2 ± 7.3	0.085	14	74.6 ± 10.1	77.5 ± 10.6	+ 1.3 ± 3.8	0.246
Central systolic BP (mmHg)	30	119.1 ± 15.2	112.7 ± 10.5	− 5.9 ± 9.1	0.008	16	118.2 ± 18.7	117.0 ± 14.4	− 1.2 ± 13.4	0.877
Central pulse pressure (mmHg)	30	42.7 ± 9.1	38.5 ± 7.7	− 4.0 ± 4.5	< 0.001	16	41.1 ± 9.0	40.8 ± 8.9	− 0.4 ± 8.0	0.776
Forward pulse pressure height (mmHg)	30	28.8 ± 6.3	26.2 ± 5.5	− 2.4 ± 3.8	0.001	16	27.9 ± 7.3	27.6 ± 7.6	− 0.3 ± 6.2	0.856
Resting pulse pressure height (mmHg)	30	17.8 ± 3.9	15.8 ± 3.5	− 1.9 ± 2.3	< 0.001	16	17.2 ± 3.8	16.3 ± 4.5	+ 3.7 ± 1.0	0.182

Data are given as mean ± standard deviation

*ABP* ambulatory blood pressure, *BP* blood pressure, *Empa* empagliflozin

Subjects treated with empagliflozin for 12 weeks showed the following changes in laboratory parameters compared to baseline: haematocrit: 40.2 ± 3.9% to 42.4 ± 3.8% (p = 0.197), HbA1c: 5.9 ± 0.6% to 5.8 ± 0.4% (p = 0.135), fasted plasma glucose: 105.9 ± 19.4 mg/dl to 95.6 ± 13.1 mg/dl (p < 0.001) and serum uric acid 6.9 ± 1.7 mg/dl to 5.7 ± 1.4 mg/dl (p < 0.001). No significant changes were found in the same clinical parameters after 12 weeks placebo therapy compared to baseline (data not shown).

### Relationship between β-OHB, BP, vascular parameters and laboratory findings (Table 3, Fig. 3)

**Table 3:** Correlations between changes in β-OHB, blood pressure and vascular parameters

	Empa Δβ-OHB	Placebo Δβ-OHB
Δ Resting peripheral systolic BP	r = 0.458	r = − 0.336
	p = 0.011	p = 0.203
Δ Resting peripheral diastolic BP	r = 0.365	r = − 0.162
	p = 0.047	p = 0.548
Δ Resting peripheral pulse pressure	r = 0.471	r = − 0.087
	p = 0.009	p = 0.749
Δ Central systolic BP	r = 0.470	r = − 0.413
	p = 0.009	p = 0.112
Δ Central pulse pressure	r = 0.391	r = − 0.266
	p = 0.033	p = 0.320
Δ Forward pulse pressure height	r = 0.451	r = 0.086
	p = 0.012	p = 0.752
Δ Resting pulse pressure height	r = 0.217	r = − 0.342
	p = 0.250	p = 0.194
Δ Systolic 24 h ambulatory BP	r = 0.321	r = 0.141
	p = 0.069	p = 0.630
Δ Diastolic 24 h ambulatory BP	r = 0.516	r = 0.195
	p = 0.002	p = 0.504
Δ Heart rate	r = 0.188	r = 0.439
	p = 0.320	p = 0.089

Δ: change between baseline and after 12 weeks of treatment, β-OHB: β-hydroxybutyrate; empa: empagliflozin; BP: blood pressure

Significant correlations were observed between the change of β-OHB and the changes in BP parameters in subjects treated with empagliflozin for 12 weeks. Greater increases of β-OHB blood levels were related to smaller decreases in diastolic ABP (r = 0.516, p = 0.002), brachial systolic (r = 0.458, p = 0.011) and diastolic (r = 0.365, p = 0.047) BP and tended to be related to smaller decreases in 24-h systolic ABP(r = 0.321, p = 0.069).

Regarding vascular parameters an increase of β-OHB correlated with the decrease of cSBP (r = 0.470, p = 0.009), cPP (r = 0.391, p = 0.033) and forward pulse pressure height (r = 0.451, p = 0.012). There were no significant correlations between changes of β-OHB and changes of BP and vascular parameters in the placebo group.

No significant correlations between changes in β-OHB and changes in the above mentioned laboratory parameters were observed.

## Discussion

To the best of our knowledge, the current study is the first to evaluate the effect of SGLT2 inhibitors on blood ketone body concentration, as assessed by β-OHB, in patients with CHF, and to analyse the relationship between changes in serum β-OHB levels and changes in vascular stiffness and BP.

### Effect of SGLT2 inhibition on serum β-OHB concentration

The main finding of this study is that 12 weeks treatment with empagliflozin leads to an increase of fasting serum β-OHB in our patients with stable CHF compared to the placebo group. Consistently, in rodent models employing empagliflozin, ketone levels were significantly increased in both lean control and obese ZSF1 groups [18], as well as in high-fat diet-fed ApoE-knockout mice [19]. In human studies with T2DM patients undergoing an acute myocardial infarction ketone bodies in the blood tended to increase in the empagliflozin group compared with the placebo group [20]. In addition, in T1DM patients ketoacidosis rate was comparable between empagliflozin 2.5 mg and placebo but increased with 10 mg and 25 mg [21]. Moreover, treatment with SGLT2 inhibitors has been demonstrated to increase blood levels of β-OHB in patients with type 1 and 2 diabetes mellitus [6, 22]. The increment in serum β-OHB concentration following SGLT2 inhibition can be explained by the SGLT2 inhibitor-induced glucosuria, which in turn accelerates the fasting state and, thereby provokes ketogenesis. Glucosuria is accompanied by osmotic diuresis and leads to volume depletion along with dehydration. Hypovolemia in combination with a carbohydrate depletion provokes the secretion of glucagon and thereby, also ketogenesis. This may explain the counterproductive effect of ketonemia on the cardiovascular system. The beneficial effect of SGLT-2 inhibitors is possibly attenuated by the increase glucagon production. These interesting effects need further evaluation. Assuming that the failing heart prefers ketone bodies as a fuel source, this might be one possible explanation for the responsible underlying mechanisms of the beneficial cardiovascular outcomes. The previously quoted “fuel hypothesis” suggests that administration of empagliflozin leads to an increase of ketone bodies, thereby optimising cardiac energy metabolism and thus, reduced cardiovascular mortality is achieved [7].

However, the increase of serum ketone bodies under SGLT2 inhibition has also been described to be linked to diabetic ketoacidosis. A recent review of literature found a small risk of diabetic ketoacidosis in patients with type 2 diabetes under the therapy of SGLT2 inhibitors. The study collective included 39 randomised controlled studies and 60,580 patients [23]. In our study we did not find any event of diabetic ketoacidosis. This is most probably due to our patient collective being comparatively small, more heterogeneous and being on a stable medication-based treatment.

### Effect of SGLT 2 inhibition on blood pressure and vascular stiffness

Analysing the effects of 12 weeks treatment with empagliflozin, we observed significant improvement in BP and vascular function. Brachial systolic BP and systolic 24 h ABP decreased significantly after 12 weeks of treatment with empagliflozin. Yet, we did not observe any significant improvement in brachial diastolic BP and 24 h ABP. Accordingly, DBP compared with SBP has been described to decrease slower in patients treated with empagliflozin [24]. Regarding vascular stiffness parameters, we observed significant improvement in cSBP, cPP, forward and resting pulse pressure heights.

### Association between serum β-OHB concentration increment and vascular parameters

In the current study we found that the improvement in vascular stiffness and BP is inversely related to the increase in serum β-OHB, indicating that a greater increase of β-OHB is associated with less improvement of central BP and 24 h ABP. Central BP and 24 h ABP are both being considered as strong independent predictors of cardiovascular events and overall mortality [25, 26].

Interestingly, our findings propose rather negative effects of the β-OHB increase after treatment with empagliflozin questioning the concept whether ketone bodies are responsible for the improved cardiovascular outcomes under the treatment with empagliflozin. Our study suggests exactly the opposite of the “fuel hypothesis”: β-OHB attenuates the beneficial effects of empagliflozin on vascular function and BP.

Coppola et al. reported increased arterial stiffness and early functional endothelial damage in children with drug resistant epilepsy that were treated with a ketogenic diet. Vascular parameters like the β-index and augmentation index, indicative for early functional endothelial damage and vascular stiffness, increased after treatment with ketogenic diet in this specific study [27]. Concordant results have been already reported. In a recent experimental study ketogenic diet aggravated hypertension in spontaneously hypertensive rats. The underlying mechanism might consist in decreasing the expression of eNOS and the endothelial marker CD31, thereby impairing endothelial-dependent vasodilatation. Furthermore, it was shown that ketogenic diet induced an increase of inflammatory parameters, as well as increased oxidative stress by the increase of reactive oxygen species [28]. On this subject, Jain et al. reported increased oxidative stress in hyperketonemic patients with type 1 diabetes [29]. These studies in accordance with our results, all indicate that ketone bodies have adverse effects on vascular function and structure. Nevertheless, overall BP decreased and vascular function improved after treatment with empagliflozin, although clearly attenuated in the patients with increased ketone bodies.

The strength of our study is that the effects of SGLT2 inhibitors on ketone bodies and their subsequent interaction with vascular parameters were observed in a double blind randomised controlled trial in patients with CHF. This is the first time that ketone bodies and their subsequent interaction with vascular parameters are evaluated in patients with stable CHF after SGLT2 inhibitor therapy and timely in face of the new guidelines for CHF.

### Limitations

Nevertheless we have to acknowledge certain limitations: our study population is relatively small which clearly diminishes the statistical power of our findings. This is also due to the fact that several subjects had to be excluded from our analysis as their serum levels of β-OHB were below the measurement range of our assay at both time points when blood was collected. However, no differences in the clinical characteristics of the subgroup with measurable β-OHB concentrations and with the total study group were found (data not shown). The lower limit of quantification for the enzymatic assay used is 0.05 mmol/l. However, we want to stress, that this assay is a validated method of β-OHB measurement used by our university laboratory in the clinical daily routine [30]. It would be useful to additionally employ a more sensitive method for future measurements.

Data on the relationship between ketone bodies, SGLT2 inhibitors and cardiovascular effects are very limited. We therefore related our data to various studies in different experimental settings, including empagliflozin-induced ketonemia, ketogenic diet and infusion with ketone bodies. It is difficult to compare and standardise blood levels of ketone bodies from different studies as they are very fluctuating and depend on various factors like length of fasting, type of diet, medications, concomitant diseases and amount of physical activity. Ketogenic diet leads to a larger increment in serum ketone body levels than SGLT2 inhibition. Accordingly, Johnson et al. observed an increase in serum β-OHB concentration of 0.24 mmol/l after 6 weeks of ketogenic diet as compared to 0.04 mmol/l in our analysis [31]. However, we were able to make similar observations like those, associated with ketogenic diet. Moreover, in our study the measurement timing and biochemichal assay was the same at both time points when blood was collected, patients were randomised in a double blind fashion and the placebo group served as a control group.

In conclusion, treatment with empagliflozin lead to a small but significant increase of serum ketones in patients with HFmEF or HFrEF. The observed improvement of BP and vascular function after treatment with empagliflozin was attenuated by the increase of serum ketone bodies.

## Data Availability

The datasets used and/or analysed during the current study are available from the corresponding author on reasonable request.

## Abbreviations

ABP: Ambulatory blood pressure
β-OHB: Beta-hydroxybutyrate
BP: Blood pressure
Δ: Changes
CHF: Chronic heart failure
cPP: Central pulse pressure
cSBP: Central systolic blood pressure
HFmEF: Heart failure with mid-range ejection fraction
HFrEF: Heart failure with reduced ejection fraction
LVEF: Left ventricular ejection fraction
SGLT: Sodium–glucose cotransporter
T2D: Type 2 diabetes mellitus
